# Elucidating Mechanisms of Toxicity Using Phenotypic Data from Primary Human Cell Systems—A Chemical Biology Approach for Thrombosis-Related Side Effects

**DOI:** 10.3390/ijms16011008

**Published:** 2015-01-05

**Authors:** Ellen L. Berg, Mark A. Polokoff, Alison O’Mahony, Dat Nguyen, Xitong Li

**Affiliations:** BioSeek, a Division of DiscoveRx Corp., 310 Utah Ave., Suite 100, South San Francisco, CA 94080, USA; E-Mails: mpolokoff@bioseekinc.com (M.A.P.); aomahony@bioseekinc.com (A.O.); dnguyen@bioseekinc.com (D.N.); xli@bioseekinc.com (X.L.)

**Keywords:** thrombosis, toxicity, primary human cells, endothelial cells, inflammation, cholesterol, aryl hydrocarbon receptor, nutrient sensing, pathway, tissue factor, bacterial sensing, hypoxia

## Abstract

Here we describe a chemical biology approach for elucidating potential toxicity mechanisms for thrombosis-related side effects. This work takes advantage of a large chemical biology data set comprising the effects of known, well-characterized reference agents on the cell surface levels of tissue factor (TF) in a primary human endothelial cell-based model of vascular inflammation, the BioMAP^®^ 3C system. In previous work with the Environmental Protection Agency (EPA) for the ToxCast™ program, aryl hydrocarbon receptor (AhR) agonists and estrogen receptor (ER) antagonists were found to share an usual activity, that of increasing TF levels in this system. Since human exposure to compounds in both chemical classes is associated with increased incidence of thrombosis-related side effects, we expanded this analysis with a large number of well-characterized reference compounds in order to better understand the underlying mechanisms. As a result, mechanisms for increasing (AhR, histamine H1 receptor, histone deacetylase or HDAC, hsp90, nuclear factor kappa B or NFκB, MEK, oncostatin M receptor, Jak kinase, and p38 MAPK) and decreasing (vacuolar ATPase or V-ATPase) and mTOR) TF expression levels were uncovered. These data identify the nutrient, lipid, bacterial, and hypoxia sensing functions of autophagy as potential key regulatory points controlling cell surface TF levels in endothelial cells and support the mechanistic hypothesis that these functions are associated with thrombosis-related side effects *in vivo*.

## 1. Introduction

Tissue factor, thromboplastin or CD142, is the cell-surface receptor for the serine protease factor VIIa and, as part of the factor VIIa-TF enzymatic complex, functions as the primary cellular initiator of blood coagulation [1,2,3,4]. The expression of tissue factor within the blood vessel lumen is tightly controlled to ensure vascular homeostasis and prevent unwanted thrombosis and occlusion of the blood vessel. Thrombosis is a normal component of wound healing, as the platelet-fibrin clots produced by coagulation recruit a variety of growth factors directly to the injured site, promoting angiogenesis and tissue repair. Under normal conditions, thrombosis is restricted to sites of tissue damage where the vessel has been denuded of endothelial cells and tissue factor is expressed on sub-endothelial smooth muscle cells or fibroblasts, or to sites of inflammation or stress where expression of tissue factor has been induced on endothelial cells and monocytes recruited from the blood. There are pathologic settings, however, where thrombosis causes severe adverse effects such as deep vein thrombosis and pulmonary embolism [2].

Untoward thrombosis or coagulopathy, and increased levels of TF are associated with a number of disease settings: cardiovascular disease conditions, such as atherosclerosis and acute coronary artery syndrome; cancer; diabetes; liver injury; and infectious diseases, such as human immunodeficiency virus (HIV), herpes simplex virus-1 (HSV-1) and Ebola virus hemorrhagic fever, which is a consumptive coagulopathy [4,5]. In addition, thrombosis-related side effects have been associated with exposure to environmental agents, such as smoking, and certain drugs including selective estrogen modulators and mTOR inhibitors [6,7,8,9]. Increased TF levels have been correlated with risk of thrombosis [10].

There is growing interest in applying *in vitro* methods to characterize the risks of side effects related to drugs and chemicals [11,12]. Physiologically relevant *in vitro* assays complementary to animal studies, provide coverage of human species specific effects, and can be used to generate high-throughput datasets that support and define adverse outcome pathways used in chemical risk assessment [13,14,15]. While data-driven approaches to build predictive classifiers are of interest, the ability to provide an in-depth understanding of toxicity mechanisms is as important, since this provides increased confidence in the predicted outcomes and potential means to mitigate adverse events.

We have been building a large chemical biology database consisting of reference chemicals and bioactive agents tested in a panel of human primary cell-based tissue and disease models, termed BioMAP systems [1,16,17,18,19,20]. These systems consist of human primary cells in complex settings including co-culture formats and/or stimulation with cocktails of factors and/or cytokines to recapitulate aspects of tissue disease states. Endpoints measured in these assays include primarily protein biomarkers that are known clinical biomarkers and disease risk factors relevant to inflammation, tissue remodeling, immune responses, hemostasis, and other biological processes. These assays have been standardized, extensively validated for reproducibility and used to test clinical stage drugs, failed drugs, tool compounds, environmental chemicals, natural products, food extracts and nanomaterials [1,16,17,18,19,20].

There are challenges in building large chemical biology datasets. In our case, the number of chemical and test agents of interest is very large, while primary human cells are expensive and can be variable. Through extensive study of the reproducibility and sources of variation in these assays, assay formats have been selected that are both informative and affordable. In the studies presented here, we have applied methods to reduce sources of variation, such as pooling cells from multiple human donors and applying plate-based normalization methods. We have also made compromises; in our screening format, although we measure a single well per endpoint, multiple concentrations per test agent and multiple endpoints in each assay are evaluated, and for each mechanism of interest, where possible, multiple agents with the same target mechanisms are tested. Replicate samples run as blinded tests for the EPA’s ToxCast program demonstrate the level of assay reproducibility [1].

These challenges are balanced by the advantages of a well-annotated large chemical biology data set. Findings with any single test agent can be immediately confirmed by evaluating the results of other test agents from the same mechanism class, or with other features in common. This data-driven approach differs from traditional hypothesis-driven research in that hypotheses are the actual outcome of the study. The value and strength of these hypotheses depend on the data that contribute to the hypothesis, the quantity and quality of the data, the number of test agents, the external information available on these agents, such as their mechanisms of action, clinical results or activities in other studies. Although this external information can be difficult to quantify, the hypotheses generated can be highly valuable, providing a framework with which to connect various findings derived from hypotheses-driven research.

The number of agents tested and mechanisms represented in this database has reached the quantity and breadth sufficient to enable compound-selective activities to be distinguished from mechanism-dependent effects. We have previously reported that selective probes for a number of target and pathway mechanisms generate signatures across a panel of 8 BioMAP systems that permit the automatic assignment of a mechanism class to new compounds [1,21]. These mechanisms include a variety of key target and pathway mechanisms of interest including those of kinase (MEK, Jak, PI3K, *etc.*), nuclear hormone receptor or NHR (glucocorticoid receptor or GR, estrogen receptor or ER, *etc.*), enzyme (HMG-CoA reductase), and G-protein coupled receptor or GPCR (Prostaglandin E receptor or EP) target classes, as well as key cellular processes such as mitochondrial and microtubule function.

These mechanism models were recently employed in a project evaluating a set of 776 compounds from the US Environmental Protection Agency’s (EPA) ToxCast^™^ program: a study in which we also demonstrated the ability to detect reproducible signatures that could be used to assign, as well as to differentiate, mechanism classes. A particularly notable finding was the identification of a common biomarker activity, increased TF, detected with compounds from two mechanism classes, specifically, AhR agonists and ER antagonists. This finding, along with the reported association of these two mechanism classes with thrombosis-related side effects, suggested that a more in-depth study of the regulation of TF in these BioMAP systems may lead to a better understanding of potential mechanisms regulating thrombosis-related side effects. We therefore undertook the present study to identify additional classes of drugs with TF-modulating activities with the goal of enabling early detection of compounds at risk for inducing thrombosis-related side effects *in vivo*.

## 2. Results and Discussion

### 2.1. Regulation of Protein Biomarker Levels in a Human Primary Endothelial Cell Vascular Inflammation Model

**Figure 1:**
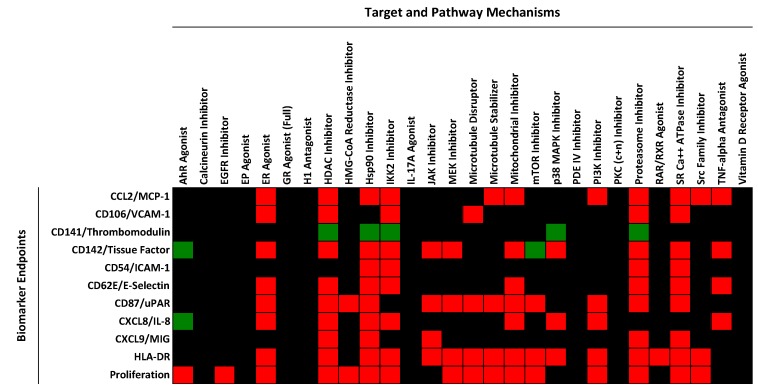
Heat map showing the effect of mechanisms on the cell surface protein expression levels of biomarker endpoints in the BioMAP 3C system. Mechanisms causing an increase in the level of the indicated biomarker are shown in green, and mechanisms resulting in decreased levels are shown as red. Black indicates that the levels were unchanged (no missing data). Data were generated from multiple compounds and an average profile representing the mechanism class was generated. The data used to generate this figure are taken from reference [21] and are included for convenience in Supplemental Table S1.

The BioMAP 3C system consists of human primary endothelial cells stimulated for 24 h with a cocktail of proinflammatory cytokines (IL-1β + TNFα + IFNγ), followed by measurement of cell surface levels of MCP-1, VCAM-1, TF, TM, IL-8, MIG, HLA-DR, E-selectin and uPAR; total protein levels (measured by sulforhodamine B (SRB) staining, see Experimental Section below and a more detailed description in Supplemental Text, Materials and Methods); and cell proliferation. We have been running this model system for over 10 years, testing various drugs, biologics and experimental tool compounds or test agents [1,16,17,18,19,20,21,22,23,24,25]. In a recent study, we reported on the evaluation of a set of 88 reference agents representing 28 pathway/mechanism classes, profiled in a panel of 8 BioMAP systems, including the BioMAP 3C system [21]. Figure 1 shows a heat map indicating the effect of each of 28 mechanism classes on the biomarker endpoint levels in the BioMAP 3C system, with significantly increased levels (>10% effect size) shown in green, and decreased levels shown in red (with no change indicated in black). As shown in this figure, each pathway mechanism evaluated in the BioMAP 3C system displays a specific pattern of biomarker changes, and correspondingly, each biomarker displays a distinguishable pattern of regulation by the various mechanism classes. Studying multiple endpoints simultaneously allows non-specific effects, for example, due to overt cell death, to be identified, preventing such confounding results from interfering with data interpretation. In Figure 1, treatment conditions leading to overt cell death were excluded prior to analysis [21].

Mechanism classes that decrease the levels of TF in this analysis (Figure 1) were found to include: inhibitors of cytokine and inflammation signaling pathways (JAK, IKK2, MEK, p38MAPK and TNF-α), inhibitors of basic cellular processes (Mitochondria, Proteasome and sarco/endoplasmic reticulum Ca^2+^-ATPase or SERCA), HDAC inhibition, and estrogen receptor (Estrogen R or ER) activation. In large part these data are consistent with previous studies demonstrating the role of NFκB, mitogen-activated protein kinase or MAPK, ER and Jak/Stat pathways in regulating TF in cells [1,26,27,28]. Although many studies on TF regulation have been performed in other cell systems, such as monocytes, mechanisms for transcriptional regulation of TF show common features [28]. See also Section 2.5 below.

### 2.2. Identification of Mechanisms Controlling Increased Levels of TF in the BioMAP 3C System

In the BioMAP 3C system, the level of TF is already elevated by cytokine stimulation (see Supplemental Text, Materials and Methods). Indeed, inhibitors of IL-1β, TNFα or IFNγ signaling all decrease TF levels in this system (Figure 1 and data not shown). Thus, identifying compounds or test agents representing diverse mechanisms that further increase TF levels presents an opportunity to study the mechanisms underlying the cell surface TF elevation and its potential pathological significance. Among the 28 mechanisms shown in Figure 1, only the mTOR inhibitor and AhR agonist classes were found to increase the levels of TF in the BioMAP 3C system. Increased TF production by indolic uremic solutes, which accumulate in chronic kidney disease, has been recently shown to be mediated by activation of the AhR pathway [29]. We had previously reported that ER antagonists also increase TF in the BioMAP 3C system, but this mechanism class was not developed as one of the 28 mechanism models [21]. In the present report we expanded our study to search a larger reference database of compound activity profiles generated in the BioMAP 3C system. This database includes previously reported profiling datasets [1,17,20], as well as various additional reference compounds and bioactive agents. Compounds included in this database were tested at 4–16 concentrations, albeit in screening format (see Experimental Section). For this analysis effort, a stringent approach was employed; only test agents that significantly increased the levels of tissue factor >30% (and having log ratio values >99% of historical vehicle control values) at two or more concentrations without causing overt cell death (see Experimental Section and Supplemental Text, Materials and Methods) were selected. The resulting list is shown in Table 1 (compounds and data sources are included in Supplemental Table S2 and experimental data tables are included in Supplemental Table S3).

**Table 1:** List of test agents that increase cell surface levels of TF in the BioMAP 3C system, their mechanism class if known, the likelihood that the indicated mechanism class is involved in the regulation of TF (as described in the text), active concentrations (in nM unless otherwise listed) and the source of experimental data. Note that data for all compounds are included in Supplemental Table S3. Test agents listed increase TF >30% at two or more concentrations as described in the Experimental Section.

Compound Name	Mechanism	Likelihood of Mechanism Involvement	Concentrations Active (nM)	Data Source
2-Mercaptobenzothiazole	AhR agonist	Probable	13,000, 40,000	Reference [1]
3-Hydroxyfluorene	AhR agonist	Probable	4400, 13,000, 40,000	Reference [1]
Benzo(b)fluoranthene	AhR agonist	Probable	4400, 13,000, 40,000	Reference [1]
C.I Solvent yellow 14	AhR agonist	Probable	13,000, 40,000	Reference [1]
FICZ	AhR agonist	Probable	0.15, 0.46, 1.4, 4.1, 12, 37, 111, 333, 1000	This study
Abiraterone	CYP17A Inhibitor	Probable	1100, 3300, 10,000	This study
Ketoconazole	CYP17A Inhibitor	Probable	3300, 10,000	This study
Clomiphene citrate	Estrogen R Antagonist	Probable	2100, 4200	Reference [1]
Histamine	H1R agonist	Probable	370, 1100, 3300, 10,000, 30,000, 90,000	This study
Histamine Phosphate	H1R agonist	Probable	1111, 3300, 10,000, 30,000	This study
Cobalt(II) Chloride Hexahydrate	HIF-1α Inducer	Probable	30,000, 100,000	This study
Tin(II) Chloride	HIF-1α Inducer	Probable	10,000, 30,000	This study
Chloroquine Phosphate	Lysosome Inhibitor	Probable	10,000, 30,000	This study
Primaquine Diphosphate	Lysosome Inhibitor	Probable	11,000, 33,000	This study
Temsirolimus	mTOR Inhibitor	Probable	0.51, 1.5, 4.6, 123	This study
Torin-1	mTOR Inhibitor	Probable	0.46, 1.4, 4.1, 12, 37	This study
Torin-2	mTOR Inhibitor	Probable	0.46, 1.4, 4.1, 12	This study
Bryolog	PKC activator	Probable	4, 12, 37, 110, 330, 1000	This study
Bryostatin 1	PKC activator	Probable	12, 37, 110, 330, 1000	This study
Bryostatin 2	PKC activator	Probable	0.15, 0.46, 1.4, 4.1, 12, 37, 111	This study
Phorbol 12-myristate 13-acetate	PKC activator	Probable	37, 41, 111, 120, 330, 370, 1000, 1100, 3300	This study
Phorbol 12,13-didecanoate	PKC activator	Probable	1.5, 4.6, 14, 41, 123, 370, 1100, 3300	This study
Picolog	PKC activator	Probable	3.7, 11, 33, 100	This study
Z-FA-FMK	Cysteine protease Inhibitor	Possible	1100, 3300, 10,000, 30,000	This study
Mifamurtide	NOD2 agonist	Possible	1111, 3300, 10,000, 30,000	This study
Ethanol	Organic Solvent	Possible	0.3%, 0.9%	This study
Oncostatin M	OSM R agonist	Possible	0.037, 0.11, 0.33, 3	This study
PAz-PC	Oxidized phospholipid	Possible	10,000, 30,000	This study
3,5,3-Triiodothyronine	Thyroid H R agonist	Possible	16,000, 32,000	Reference [1]
Concanamycin A	Vacuolar ATPase Inhibitor	Possible	0.14, 0.41, 1.2, 3.7, 11, 33, 100	This study
MK-2206	AKT Inhibitor	Unknown	1111, 3300, 10,000	This study
Crizotinib	ALK, c-met Inhibitor	Unknown	1111, 3333	This study
*N*-Ethylmaleimide	Alkylating agent	Unknown	37, 110	This study
Terconazole	Anti-fungal	Unknown	9300, 9400	This study
GDC-0879	B-Raf Inhibitor	Unknown	370, 1100	This study
KN93	CAMKII Inhibitor	Unknown	1100, 3300	This study
8-Hydroxyquinoline	Chelating agent	Unknown	18, 55	Reference [1]
Linoleic Acid Ethyl Ester	Fatty Acid	Unknown	10,000, 30,000, 90,000, 270,000	This study
Tris(1,3-dichloro-2-propyl) phosphate	Flame retardant	Unknown	13,000, 40,000	Reference [1]
Fenaminosulf	Fungicide	Unknown	13,000, 40,000	Reference [1]
Mancozeb	Fungicide	Unknown	20,000, 40,000	Reference [1]
Primidone	GABA R agonist	Unknown	1500, 4400	This study
Mometasone furoate	GR agonist	Unknown	1111, 3300	This study
Desloratadine	H1R antagonist	Unknown	10,000, 30,000	This study
A 205804	ICAM, E-selectin inhibitor	Unknown	41, 123	This study
Dodecylbenzene	Industrial chemical	Unknown	1200, 2500	Reference [1]
UO126	MEK Inhibitor	Unknown	4.6, 14, 41	This study
Imatinib	PDGFR, c-Kit, Bcr-Abl Inhibitor	Unknown	3300, 10,000	This study
ZK-108	PI-3K Inhibitor (βγ-selective)	Unknown	1100, 3300, 10,000	This study
GW9662	PPARγ agonist	Unknown	7400, 22,000	This study
PP3	SRC Kinase Inhibitor	Unknown	3300, 10,000, 30,000	This study
TX006146	Unknown	Unknown	13,000, 40,000	Reference [1]
TX006237	Unknown	Unknown	1500, 4400	Reference [1]
TX011661	Unknown	Unknown	5000, 10,000, 20,000	Reference [1]
U-73343	Unknown	Unknown	560, 1700	This study

Test agents that increase cell surface levels of TF in the BioMAP 3C system were annotated for their known mechanisms and for the likelihood that their reported mechanism of action is responsible for the increased TF (Table 1). Test agents for which multiple agents within the same mechanism class were identified (strong evidence suggesting that the increase in TF is mechanism related), were designated as highly likely or “probable”. Mechanism classes for which this confidence qualifier applied include AhR agonists, Estrogen R antagonists, CYP17A inhibitors, agonists of histamine receptors (H1R agonists), mTOR inhibitors, PKC activators, lysosomal inhibitors, and metal-containing inducers of hypoxia (HIF-1α inducers), Co(II)Cl_2_ (cobalt chloride) and Tn(II)Cl_2_ (stannous chloride). Mechanism classes for which only a single agent within that mechanism class was tested were designated as “possible”. These mechanisms include agonists of NOD2 (mifamurtide), oncostatin M receptor, (OSMR) and thyroid hormone (TR) receptor (3,5,3-triiodothyronine); ethanol, the oxidized LDL-derived species, PAz-PC; and the cysteine protease inhibitor, Z-FA-FMK. Induction of TF on smooth muscle cells by oncostatin M has been previously reported [30]. In some cases, we identified agonist/antagonist agent pairs that produce opposite effects on TF and provides additional confidence for mechanism involvement (Table 2). These mechanisms include AhR, ER, PKC and H1R. Test agents for which there is good evidence that the effect on TF is due to their reported mechanisms are considered to be most informative for understanding mechanistic regulation of TF. These agents (listed as “probable” or “possible” in Table 1) were then analyzed for known pathway mechanisms, common biological processes, as well as for association with thrombosis-related biology.

### 2.3. Evidence for the Role of Autophagy in Controlling Cell Surface TF Levels

**Figure 2:**
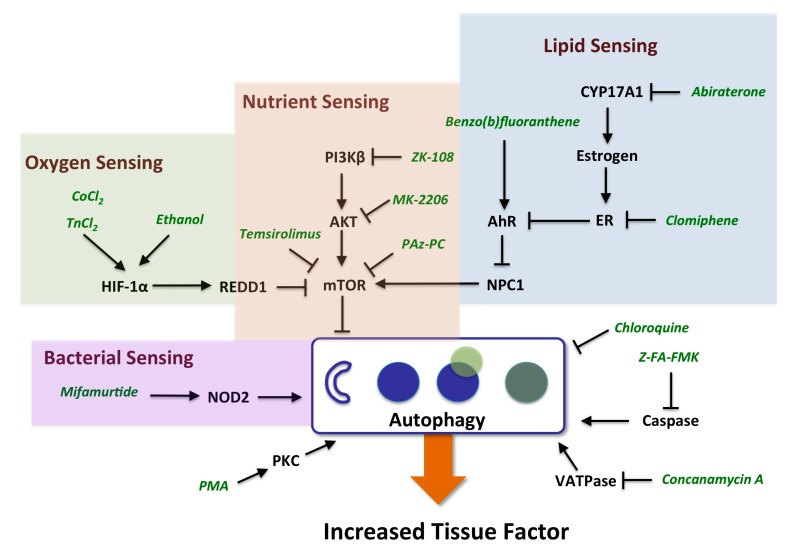
Schematic representing pathway mechanisms that regulate the cell surface expression of TF. Mechanisms identified in the current study that increase TF in the BioMAP 3C system and regulate autophagy by impacting nutrient, oxygen, lipid or bacterial sensing functions as detailed in the text. The process of autophagy involves nucleation of autophagosomes, engulfment of cellular constituents, fusion with lysosomes, and subsequent degradation of the contents.

Strikingly, the mechanisms identified as regulating the increase in TF (listed as “probable” or “possible” in Table 1) are known to regulate the process of autophagy (Figure 2). Autophagy involves the engulfment of cytoplasmic constituents, including lipids, cellular organelles and protein aggregates into autophagosomes which initially fuse with late endosomes, and subsequently with lysosomes to form autolysosomes wherein the engulfed materials are degraded and subsequently exported back into the cytoplasm or to mitochondria where they can be utilized in energy production [31]. Autophagy is initiated by autophagy-related gene products, Atg proteins, that together with phosphatidylethanolamine (PE, the second most abundant lipid in mammalian membranes) and complexes containing ULK1 kinase and Beclin1, initiate the formation of autophagosomes. Autophagy is an adaptive process that increases with nutrient deprivation in order to maintain energy homeostasis and also participates in the removal of damaged cellular organelles and constituents, including aggregated proteins or engulfed bacteria. Autophagy plays a role in lipid metabolism, insulin sensitivity, immune responses and cell death, and can be induced by various cellular stresses in addition to nutrient deprivation, such as hypoxia, heme exposure and infection. Autophagy has also been shown to play an important role in liver physiology and pathology [32]. In neutrophils from septic patients, TF has been shown to co-localize with autophagosomes, in a process that involves autophagy and mediates delivery of TF to extracellular structures known as neutrophil extracellular traps (NETs). NETs have been implicated in the pathogenesis of bacterial sepsis including thrombotic sequelae [33]. The mechanisms represented in Table 1 that increase TF levels in the 3C system are largely known to affect autophagy by increasing the number of autophagosomes either by stimulating autophagosome formation, or by preventing their maturation or subsequent degradation of their cargo, for example, through inhibition of lysosomal function. Autophagy plays a hemostatic control function inside the cell, and is subject to regulation by nutrient, oxygen, bacterial, and lipid-sensing mechanisms, involving key regulators mTOR, HIF-1α, NOD2 and NPIC-1, as discussed below.

**Table 2:** List of target agonist and antagonist pairs that modulate cell surface levels of TF in the BioMAP 3C system in a reciprocal manner. Test agents listed increase TF at two or more concentrations as described in the Experimental Section, or significantly decrease the level of TF as indicated.

Target Class	Action	Compound	TF Level	Reference
AhR	Agonist	2-Mercaptobenzothiazole	Increased	This study
AhR	Agonist	3-Hydroxyfluorene	Increased	This study
AhR	Agonist	Benzo(b)fluoranthene	Increased	This study
AhR	Agonist	C.I Solvent yellow 14	Increased	This study
AhR	Agonist	FICZ	Increased	Reference [21]
AhR	Antagonist	CH223191	Decreased	Unpublished, 2014
ER	Antagonist	Clomiphene citrate	Increased	This study
ER	Agonist	17α-Ethynylestradiol	Decreased	Reference [21]
ER	Agonist	17β-Estradiol	Decreased	Reference [21]
ER	Antagonist	Tamoxifen	Increased	Reference [1]
ER	Antagonist	Tamoxifen Citrate	Increased	Reference [1]
ER	Antagonist	Fulvestrant	Increased	Reference [1]
ER	Antagonist	Raloxifene hydrochloride	Increased	Reference [1]
H1R	Antagonist	Astemizole	Decreased	Reference [21]
H1R	Antagonist	Ketotifen Fumarate	Decreased	Reference [21]
H1R	Antagonist	*trans*-Triprolidine	Decreased	Reference [21]
H1R	Agonist	Histamine	Increased	This study
H1R	Agonist	Histamine Phosphate	Increased	This study
PKC	Activator	Bryolog	Increased	This study
PKC	Activator	Bryostatin	Increased	This study
PKC	Activator	Bryostatin 1	Increased	This study
PKC	Activator	Phorbol 12-myristate 13-acetate	Increased	This study
PKC	Activator	Phorbol 12,13-didecanoate	Increased	This study
PKC	Activator	Picolog	Increased	This study
PKC	Inhibitor	GF 109203X	Decreased	Reference [21]
PKC	Inhibitor	Go 6983	Decreased	Reference [21]
PKC	Inhibitor	Ro-32-0432	Decreased	Reference [21]

#### 2.3.1. mTOR and HIF-1α Play a Key Role in Autophagy

mTOR is a key negative regulator of autophagy initiation and well-known to be inhibited under conditions of nutrient deprivation [34]. mTOR regulates autophagy through the mTOR complex 1, mTORC1, which inhibits the function of Atg kinases within the UNC-51-like kinase or ULK and Beclin 1 complexes, thereby controlling the initiation of autosome nucleation. Inhibition of mTOR leads to increased formation of autophagosomes [35]. Of interest, this process has been associated with thrombosis, as the highly selective mTOR inhibitor, rapamycin, has been shown to promote thrombosis through a mechanism of membrane remodeling involving the formation of membrane ruffles and microvilli structures in endothelial cells in a process that could be inhibited by 3-methyladenine, which blocks autophagosome formation [36]. mTOR and HIF-1α comprise a functional switch in regulating hypoxia-induced autophagy in endothelial cells [37]. CoCl_2_ and stannous chloride, metal inducers of HIF-1α, and both of which strongly increase cell surface levels of TF in the BioMAP 3C system, are known to promote autophagy. CoCl_2_ exposure induces REDD1, a HIF-1α-induced target gene that is a critical regulator of mTOR signaling during hypoxic stress. REDD1 inhibits mTOR by stabilizing the tuberous sclerosis 1-2 or TSC1-TSC2 inhibitory complex for mTOR [38,39].

#### 2.3.2. AhR, Lipid Metabolism and Sterol Sensing in Autophagy

The process of autophagy also plays a key role in regulating the metabolism of lipids, including cholesterol. AhR ligands have also been shown to regulate lipid metabolism and cholesterol biosynthesis, and the sterol-sensing transmembrane protein, NPC1, has been shown to be critically involved [40]. In endothelial cells, cholesterol trafficking via NPC1 has been shown to be required for mTOR function [41]. NPC1 is involved in the intracellular transport of cholesterol to post-lysosomal destinations, and loss-of function mutations in the NPC1 gene are associated with Niemann-Pick disease, type C, in which lipid products accumulate in late endosomes and lysosomes. Patients with this disease present with neurological symptoms and liver cholestasis. NPC1 gene knockout mice have been characterized by defects in autophagic flux, with increased numbers of autophagosomes, but impaired degradation of autolysosomes [42]. Mice lacking NPC1 are also predisposed to atherothrombosis in ApoE-knock-out models of atherosclerosis [43]. In human macrophages, AhR activation has been shown to result in lipid accumulation with a cellular phenotype similar to that of NPC1 knockdown by siRNA. This response is dependent on AhR and can be rescued by NPC1 overexpression [40]. Our finding that TF levels are increased both by AhR agonists as well as mTOR inhibitors is consistent with a role for NPC1 and the autophagy process in the regulation of cell surface TF levels. Regulation of TF by ER antagonists may also be related to this mechanism. Estrogen receptors are known to regulate AhR function in a reciprocal fashion and our data are consistent with this. In the BioMAP 3C system, AhR agonists and ER antagonists increase the level of TF, while ER agonists and CH223191, an AhR antagonist, reduce the cell surface levels of TF ([1] and X.T., unpublished observations, see also Table 2). Furthermore, increased TF levels were observed with the sterol synthesis inhibitors, ketoconazole and abiraterone, which inhibit CYP17A1 (steroid 17-α-monooxygenase), a key enzyme in the steroidogenic pathway and for the synthesis of estrogen. This mechanism could affect TF levels similar to ER antagonists, involving AhR and NPC1 and the autophagy process.

#### 2.3.3. Autophagy-Dependent Bacterial Sensing Mechanisms Are Involved in the Regulation of TF

The involvement of sensing mechanisms for oxygen (through HIF1-α), nutrient (mTOR), and lipid (through AhR/NPC1) by the autophagy system suggests that other autophagy-related sensing mechanisms may also regulate TF. Indeed, mifamuritide, a synthetic derivative of the mycobacterial product muramyl dipeptide, and agonist of NOD2, also increased TF levels in the BioMAP 3C system. NOD2 is an intracellular pattern recognition receptor and bacterial sensor protein involved in immune responses. NOD2 has been shown to direct autophagy by recruiting the autophagy protein ATG16L1 to the plasma membrane to promote engulfment of bacteria [44].

#### 2.3.4. Other Mechanisms of Interest

PKC activation is another mechanism that increased levels of TF in the BioMAP 3C system and has also been shown to induce autophagy [45]. PKC activation is known to increase and stabilize HIF-1α protein levels, and PKC inhibitors have been shown to block hypoxia mediated transactivation of HIF-1α-responsive promoters [46]. Interestingly, the PKC inhibitor GF109203X employed in these studies, has the opposite effect of PKC activators in the BioMAP 3C system, decreasing the levels of TF (Table 2 and [21]). The increase in TF by histamine, oncostatin M (OSM) and thyroid hormone (TR) receptor agonists suggests that these pathways may also be of interest in exploring the connection of autophagy to thrombosis-related biology. Histamine has been shown to induce TF in human aortic endothelial and vascular smooth muscle cells, and this activity is blocked by MAPK inhibitors, similar to the results observed in the present study [47]. The mechanism by which OSM increases TF may be through AhR, as OSM has been shown to increase AhR levels [48]. OSM also increases HIF-1α protein levels by increasing mRNA transcription via Jak/Stat3 and MAPK pathways [49]. Both of these possibilities affect autophagy and OSM has been shown to be involved in the regulation of processes such as liver development and regeneration, hematopoiesis, and angiogenesis. Thyroid hormone is known to stimulate fatty acid β-oxidative metabolism by inducing autophagy to deliver fatty acids to the mitochondria [50]. Interestingly elevated thyroid hormone levels (free thyroxine) are associated with a procoagulant state, and have been found to be a strong risk factor for venous thrombosis in a large population-based prospective study [51]. PAz-PC, 1-palmitoyl-2-azelaoyl PC, is an active species derived from oxidized low-density lipoprotein or LDL [52]. Oxidized LDL is generated in lipid-rich atherosclerotic plaques, activates HIF-1α and promotes angiogenesis [53]. In human endothelial cells, oxidized LDL activates the autophagic lysosome pathway [54]. The increase in TF by ethanol may also be related to the role of autophagy in TF regulation. Ethanol has been shown to increase the autophagic vacuole content in liver cells and the induction of autophagy has been proposed to contribute to the hepatotoxicity of alcohol [55,56]. Figure 2 summarizes the relationship of the mechanisms identified in the present study as leading to increased cell surface TF levels and their association to the process of autophagy.

In some cases (Table 1), only a single compound from a mechanism class was identified as causing an increase in TF levels whereas other compounds from the same class did not (Table 2, agents for which confidence in mechanism is listed as unknown). In these cases, the increase in TF may be due to secondary or off-target activities. Inhibitors of 5-hydroxytriptamine receptor or 5-HTR, MET proto-oncogene, receptor tyrosine kinase or c-met, cholesterylester transfer protein or CTEP, fibroblast growth factor receptor or FGFR, histamine receptor 1 or H1R, mitogen-activated protein kinase kinase or MEK, platelet-derived growth factor receptor or PDGFR, and agonists of gamma-aminobutyric acid receptor or GABA R, GR, and peroxisome proliferator-activated receptor-γ or PPARγ fall into this group. Alternatively, it is also possible that the increased TF by these compounds is target-related, but off-target activities shared by the other compounds in that mechanism class mask the effect. Further analysis of these compounds will be needed to distinguish these possibilities. This illustrates one of the challenges of chemical biology approaches: specifically, that many test agents, particularly small molecules, kinase inhibitors and GPCR compounds, are known to interact with multiple targets, thus caution must be used for the interpretation of activities found. Our approach attempts to mitigate this problem by focusing on the pathway interpretation of only those test agents for which we have higher confidence that the observed activities are mechanism related. However, while results with this group of test agents may not be informative as to the mechanistic understanding of TF regulation, such findings may be useful in explaining compound specific effects *in vivo*.

### 2.4. Advantages and Limitations of Chemical Biology Approaches

One of the advantages of data-driven chemical biology approaches is that data sets with large numbers of replicates or compound exemplars can help promote confidence in the results. For example, the finding that a number of AhR agonists at multiple concentrations all significantly increase the level of TF increases the confidence that AhR is mechanistically involved. In addition, as demonstrated in the present study, given sufficient target and mechanism coverage, useful and interesting pathway models that connect a large number of targets can be constructed. Such data-driven and hypothesis-free approaches can advance knowledge in new directions outside current focus areas of research.

A significant challenge for large scale *in vitro* studies, however, is the number of possible systems, cell types, and culture conditions that can be utilized. It is imperative, that the cell culture methods be as consistent as is possible when testing large numbers of agents, otherwise the results cannot be comparable. Thus, in the present study, care was taken in operating the BioMAP 3C cell culture model, having a quality management system in place, with the attendant procedures set for cell qualification, reagent validation, standard operating procedures (SOPs), staff training, and assay performance criteria (see Supplemental Text, Materials and Methods). It is also desirable to use *in vitro* systems that are more physiologically relevant. In the present study, this was first accomplished by using early passage human primary endothelial cells where their physiological response to stimulation is preserved and, in contrast to cell lines, are more representative of cells *in vivo*. Secondly, the cell culture environment contains a cocktail of proinflammatory cytokines, a setting that is relevant for vascular inflammation settings *in vivo*. Given that humans, unlike experimental mice, are exposed to a wide range of environmental insults including infectious agents, bacterial products, and pollution, these conditions may be more reflective of studies in human populations than in-bred, experimental mouse studies. That the immune system in humans can be characterized as activated, *versus* resting in mice is well known and supported by differences between mouse and man [57]. We have previously reported on the robust nature of this system, the results of which suggest that we have captured so-called systems behavior [58]. Finally, in the present study, assessing cell surface levels of TF protein, rather than TF mRNA levels, may explain why we were able to identify novel mechanisms for regulation.

There are limitations to chemical biology approaches as they require the availability of sufficient numbers of chemical or biological probes and a good understanding of their target selectivity. The assays as performed provide a snapshot in time, integrating multiple levels of mechanistic regulation (transcription, translation, cellular localization, *etc.*), so can be difficult to deconvolute. Thus, further understanding of hypotheses that are generated require additional types of studies, for example using knockdown or combination studies. In addition, the current study does not address functional aspects of the increased TF that is measured, such as enzymatic activity or signaling through interactions with protease-activated receptors [59,60].

### 2.5. Relationship between Tissue Factor Expression and Function

There are somewhat limited data showing TF expression on endothelial cells *in vivo* [28]. Several of the studies that have successfully shown TF expression on endothelial cells have relied on co-localization with the endothelial cell marker, CD31 [61,62]. Most studies have been performed in models of endotoxemia using *E. coli* or lipopolysaccharide, which are acute stimulators of inflammation, whereas the conditions represented in the present study could be considered more relevant to chronic inflammatory settings. Nonetheless, the present results do demonstrate an association between chemicals and drugs that increase TF levels in cytokine-stimulated endothelial cells and incidence of thrombosis related side effects in humans. While a functional role for endothelial cell TF in these side effects remains to be established, the association of increased TF in the BioMAP 3C system with the potential for thrombosis does suggest that this assay may have predictive value. The process of thrombosis is also important for wound healing and repair. Thus, conditions that promote this may also be associated with pro-thrombotic states. Also, although speculative, in conditions of nutrient deprivation, in which there is induction of autophagy, an increase in the level of endothelial cell TF could potentially play a role in the recruitment of nutrient-rich platelets to the tissue site.

The procoagulant function of TF is known to be regulated at multiple levels. Expression at the cell surface is itself regulated by transcription, translation and post-translational processing, as well as potentially by the mechanisms described herein involving vesicular cycling controlled by the process of autophagy. In order to promote thrombosis, TF binds to factor VIIa and the resulting complex, TF-VIIa, then proteolytically cleaves and activates Factor X to form the TF-VIIa-Xa complex, which in turn, activates thrombin to initiate clot formation and also cell signaling through protease-activated receptors (PARs) [63]. Expression of TF is not sufficient for maximal procoagulant activity as further stimulation (induction of calcium flux, protease treatment) increases this process. A variety of mechanisms have been proposed to regulate the procoagulant activity of TF at the cell surface including dimerization, association with lipid rafts, disulfide bond formation, and phosphatidyl serine exposure [64]. These mechanisms of encryption and decryption of tissue factor may play an important role in regulating TF function *in vivo* [65].

The current study successfully illustrates, however, the utility of this approach in generating novel hypotheses, in bridging disparate sources of information, from biochemical, to *in vitro* and *in vivo* studies, as well as bringing together multiple pathways into a larger framework model. In the case presented here, the association of the various mechanisms found to increase endothelial cell surface TF levels with the process of autophagy, and in particular, with increased numbers of autophagic vacuoles, now can be connected with pathologic findings, as effects on autophagic vacuole formation can present as a histologically defined feature. The connection of these data to clinical outcomes, *i.e.*, an increase in thrombosis, then provides the anchor point on which an adverse outcome pathway framework is built.

### 2.6. Applications for Risk Assessment and Adverse Outcome Pathways

There has been a significant effort over the last several years to advance the science of chemical risk assessment by incorporating high-throughput *in vitro* screening data, exemplified by the EPA’s ToxCast™ program, the Tox21 initiative supported by the National Institutes of Health (NIH), Environmental Protection Agency (EPA) and Food and Drug Administration (FDA), and other efforts [1,11,12,20,66,67]. The predictive models developed as a part of these initiatives rely on a battery of assays. Indeed, a recently proposed method to predict estrogenic responses for chemical prioritization uses a panel of 13 *in vitro* assays that interrogate multiple endpoints related to estrogen receptor signaling [68]. *In vitro* assays can also support risk assessment by improving the understanding of toxicity mechanisms or potentially by contributing to predictive models that provide quantification of risk. Indeed, the present study illustrates the use of these data to elucidate such toxicity mechanisms. This effort required significant efforts in data curation and annotation, and reliance on having multiple compounds and replicates to build confidence in the results. Indeed, the pathway scheme shown in Figure 2, may find utility as a framework for building Adverse Outcome Pathways (AOPs), connecting the indicated target mechanisms (AhR, mTOR, *etc.*) through their effects on TF, to measurable clinical outcomes, such as one or more thrombosis-related side effects.

The use of these *in vitro* data for quantification of risk will require additional types of studies and information. In order to evaluate any predictive value of measuring TF levels in the BioMAP 3C system, validation studies will be needed that incorporate *in vivo* exposure data, and meet higher standards for data reproducibility, in order to achieve sufficient confidence in the results. Human exposure information is often not readily available, and is it not straightforward to predict *in vivo* exposures based on compound concentrations tested *in vitro*. While advances have been made in this area [69], the incorporation of population variations in drug metabolism into risk assessment remains a challenge.

## 3. Experimental Section

### 3.1. Materials and Reagents

A detailed description of the materials, reagents and methods are included in the Supplemental Text, Materials and Methods. Compounds and sources are listed in Supplemental Table S2*.*

### 3.2. Cell Culture Methods

Preparation and culture of early passage primary human endothelial cells (Cell Applications, Inc., San Diego, CA, USA), from 3–5 pooled donors at passage 4 and methods for the BioMAP 3C system was as previously described [1,17,20], and detailed in the Supplemental Text, Materials and Methods section. Primary human cells utilized in this work were obtained under protocols that were reviewed by the Institutional Review Board(s) (IRB) that operate in accordance with the requirement of EPA Regulation 40 CFR 26 and HHS Regulation 45 CFR 46 of the US Federal Government for the protection of human research subjects. The following concentrations/amounts of agents were added to confluent cells: IL-1β, 1 ng/mL; TNF-α, 5 ng/mL; IFN-γ, 20 ng/mL.

### 3.3. Preparation of Test Agents

Test agents were prepared in DMSO (small molecules) or PBS (biologics) and added at indicated concentrations to confluent cells. Test agents were added 1 h before stimulation of the cells, and were present during the subsequent 24 h period. Final DMSO concentration was <0.1%–0.2%. Positive control samples including colchicine, 1.1 μM, and non-stimulated samples were included as controls on every plate. Vehicle controls were tested at 6 or more replicates per plate.

### 3.4. Endpoint Measurements

The cell surface levels of readout parameters were measured by cell-based ELISA and other assessments as described [1,17,20] (and see Supplemental Text, Materials and Methods). We have validated the mRNA expression of TF on stimulated HUVEC by microarray (data not shown). Across experiments (more than 600), ELISA optical density or OD (450–650) values for TF were 0.41 ± 0.14 (mean and standard deviation or SD) for the stimulated condition and 0.22 ± 0.07 for the non-stimulated condition (control antibody OD values were 0.082 ± 0.027). The log_10_ ratio of the non-stimulated *versus* the stimulated condition was −0.30 ± 0.01. Briefly, microtiter plates were treated, blocked, and then incubated with primary antibodies or isotype control antibodies (0.01–0.5 μg/mL) for 1 h. After washing, plates were incubated with a peroxidase-conjugated anti-mouse IgG secondary antibody or a biotin-conjugated anti-mouse IgG antibody for 1 h followed by streptavidin-horseradish peroxidase for 30 min. Plates were washed and developed with 3,3',5,5'-Tetramethylbenzidine substrate and the absorbance (OD) was read at 450 nm (subtracting the background absorbance at 650 nm). Overtly adverse effects of compounds on cells were determined by measuring alterations in total protein using 0.1% sulforhodamine B (SRB) staining of cells after fixation with 10% trichloroacetic acid, and reading wells at 560 nm [70].

### 3.5. Statistical Analysis

Statistical methods for BioMAP profile data have been described previously [1,17,20]. Measurement values for each endpoint in a treated sample are divided by the mean value from at least six vehicle control samples (from the same plate) to generate a ratio. All ratios are then log_10_ transformed. Significance prediction envelopes are calculated from historical negative control samples tested (e.g., 95%). Given the control distribution for each system-readout combination, the significance of an individual readout ratio can be computed from the empirical distribution by taking the 95th percentile as compared to the control ratios. Overtly cytotoxic compounds are identified as those that reduce the levels of total protein (sulforhodamine blue, SRB) below 50% (having a log_10_ ratio of SRB <−0.3). The 95th percentile for historical controls for endpoints in the BioMAP 3C system ranged from log_10_ ratio values of ±0.013 (Mig) to ±0.069 (Proliferation). The 95th percentile historical control range for TF is ±0.058. To confidently identify compounds that increase the level of TF, only test agents for which profiles showed an increase in the level of TF more than 30% (log_10_ ratio values >0.12) at 2 or more concentrations were selected.

### 3.6. Data Sources

Data employed in the present study are a combination of previously published work [1] and new data. Sources of data are listed in Table 1 and also in Supplemental Table S2.

## 4. Conclusions

Here we describe a large-scale chemical biology analysis of the regulation of cell surface TF in cytokine-stimulated primary human endothelial cells, a model of vascular inflammation, using well-defined chemical and biological probes. This study identified the process of autophagy and its function in bacterial, nutrient, oxygen and lipid sensing as a key point of regulation. These data also support a role for autophagy in the regulation of thrombosis and thrombosis-related side effects. Many of the mechanisms identified have been associated with thrombotic side effects, such as deep vein thrombosis or pulmonary embolism.

TF has complex regulation, and indeed a functional role on endothelial cells has been somewhat controversial. TF is regulated not only at the transcriptional level, but posttranslationally, including potential cycling between cryptic forms. The studies here do not address TF enzymatic or signaling functions, but the results are consistent with cell surface levels of TF in our system and correlative data with *in vivo* thrombosis potential.

The present study also provides the ability to now connect molecular targets and pathway mechanisms through their effects on TF to thrombosis-related side effects. In this way, these data can be used to support a framework for building adverse outcome pathways for thrombosis-related side effects, anchored at the molecular target on one end and connected through these mechanisms to TF and directly to the clinical effects.

## Supplementary Materials

Supplementary materials can be found at: http://www.mdpi.com/1422-0067/16/01/1008/s1.
